# DE-RISKING THE EMPLOYER-EMPLOYEE RELATIONSHIP WHEN WORKERS HAVE MILD COGNITIVE IMPAIRMENT OR YOUNG ONSET DEMENTIA

**DOI:** 10.1093/geroni/igac059.496

**Published:** 2022-12-20

**Authors:** Josephine McMurray, Arlene Astell, AnneMarie Levy, Jennifer Boger, Catherine Burns

**Affiliations:** Wilfrid Laurier University, Waterloo, Ontario, Canada; University of Toronto, Toronto, Ontario, Canada; Wilfrid Laurier University, Waterloo, Ontario, Canada; Waterloo University, Waterloo, Ontario, Canada; Waterloo University, Waterloo, Ontario, Canada

## Abstract

The employee-employer relationship is one that is well established in law and involves a contract between a worker who performs the work, and an employer who determines when, how and where the work is done. Many countries have introduced legislation that helps guide employee-employer behavior when workers’ performance of their duties is disrupted by illness or disability, such as mild cognitive impairment or young onset dementia (MCI|YOD). At any one time, about 8-12% of the workforce is off due to occupational or non-occupational work injury or illness with direct disability and absence costs projected about 7% of payroll in 2000. Drawing from data gathered in a systematic literature review and case study, this paper determined that uncertainty and risk influence employer-employee relationships when workers are identified with MCI|YOD. Employers’ industry context, size and corporate values influence their “integrated risk management” approach to workers with MCI|YOD, and early intervention is considered key to managing financial and liability risk. In contrast, progressive onset, delayed diagnosis, stigma, privacy concerns, workplace uncertainty, and lack of information may delay employee disclosure, a common employee risk mitigation strategy. We conclude by considering strategies to de-risk relationships between workers with MCI|YOD and employers using dual process theory to understand intuitive and rational decisions in the context of the disability management process.

